# Enhancing epidemic preparedness in Somalia: Lessons from mpox outbreaks in East Africa

**DOI:** 10.1016/j.jtumed.2024.12.004

**Published:** 2024-12-12

**Authors:** Fartun Sharif Mohamed, Olalekan John Okesanya, Mohamed Mustaf Ahmed, Abdiwali Ahmed Hirey, Bashiru Garba, Abdirasak Sharif Ali

**Affiliations:** a Blood Bank Unit, Banadir Hospital, Mogadishu, Somalia; b Department of Medical Laboratory Science, Neuropsychiatric Hospital Aro, Abeokuta, Nigeria; c Department of Medicine and Surgery, Faculty of Medicine and Health Sciences, SIMAD University, Mogadishu, Somalia; d Department of Public Health, Faculty of Medicine and Health Sciences, SIMAD University, Mogadishu, Somalia; e Department of Microbiology and Laboratory Sciences, Faculty of Medicine and Health Sciences, SIMAD University, Mogadishu, Somalia

To the editors of Journal of Taibah University Medical Sciences:

The mpox outbreak, once confined to Central and West Africa, has now emerged as a significant global public health concern, with an increasing number of cases, particularly in East Africa.^1^ Similar patterns of mpox outbreaks have been observed in neighboring East African countries, such as Kenya, Rwanda, Burundi, and Uganda. Kenya reported its first Mpox case in July 2024, and by mid-August, 14 suspected cases had been identified, with Burundi experiencing rapid spread of the outbreak. As of August 2024, there were 545 suspected cases and 142 confirmed cases across the region. Mpox outbreaks have also been reported in Rwanda and Uganda, with confirmed cases linked to travel to the Democratic Republic of the Congo (DRC). Notably, no mpox cases have been reported in Somalia, highlighting the cross-border nature of disease spread. Genomic sequencing has revealed that these cases are linked to clade I of the mpox virus, further complicating regional response efforts.^2^^,^^3^

Three decades of civil war and instability, coupled with natural disasters such as droughts and floods, have weakened Somalia's health systems and contributed to it having some of the lowest health indicators in the world. Of the country's 15 million people, 26–70% live in poverty, depending on the region, and an estimated 2.6 million people have been internally displaced.^1^^,^^2^ The country's universal health coverage (UHC) index is 25 out of 100.^4^ However, recent efforts to rebuild and strengthen the health system have shown promise, particularly in improving primary healthcare coverage in urban areas.^5^

To prepare for a potential mpox outbreak, Somalia can implement several key strategies with a primary focus on strengthening surveillance systems. Enhancing disease surveillance networks across the country is essential for early detection and response to mpox cases, including improving reporting mechanisms and integrating community-based surveillance. In addition, providing specialized training to healthcare workers on mpox diagnosis, treatment, and infection control measures is vital for effective outbreak management**.** Upgrading laboratory facilities and enhancing testing capabilities for accurate and timely Mpox diagnosis are also critical for confirming cases and monitoring disease spread.^6^ As a low-income country, Somalia faced unique challenges in preparing for and responding to outbreaks. These include limited financial resources, inadequate health care infrastructure, and competing health priorities. Additionally, the country's large nomadic population and areas with limited government control pose challenges to comprehensive health coverage and outbreak responses.^7^

Regional cooperation in East Africa is crucial for effective disease control and Somalia can benefit from and contribute to these efforts. The East African Community (EAC) has established mechanisms for cross-border disease surveillance and response, and Somalia could potentially join or collaborate with the EAC to enhance its preparedness.^8^ Additionally, participation in regional initiatives such as the Africa CDC's Regional Collaborating Centers would provide Somalia with valuable resources and expertise to strengthen its health security.^9^

Key recommendations for policymakers, health officials, and international organizations to support Somalia's preparedness efforts include investing in strengthening the country's primary healthcare system, particularly in rural and hard-to-reach areas. Developing a comprehensive national action plan for health security that includes specific measures for Mpox preparedness and response is crucial. Providing technical and financial support for the establishment of a robust national public health institute capable of coordinating disease surveillance and response activities is highly recommended.^6^ Furthermore, investing in training programs for healthcare workers, laboratory technicians, and epidemiologists will help build a skilled workforce capable of managing potential outbreaks.^10^

In conclusion, while Somalia has not yet reported any mpox cases, its fragile healthcare system faces significant challenges in managing potential outbreaks**.** Strengthening disease surveillance, training healthcare workers, and enhancing laboratory capacity are essential steps toward improving preparedness. Collaboration with regional bodies such as the EAC and Africa CDC can provide crucial resources and support**.** By addressing these challenges and reinforcing its health system, Somalia can enhance its preparedness for future mpox outbreaks and improve its overall health security.
